# Supplementary material for: Identification of photoperiod-induced specific miRNAs in the adrenal glands of Sunite sheep (Ovis aries)
Source: Front Vet Sci. 2022 Jul 22;9:888207. doi: 10.3389/fvets.2022.888207 (PMC9354845; doi:10.3389/fvets.2022.888207)
Supplement: Supplementary file 1 [file Data_Sheet_1.ZIP › Supplementary file/miRDeep2 file.zip!/miRDeep2 file/Novel_Structure/SPLP42A2/Novel_586.pdf]

Provisional ID	:	NC_019463.2_16570
Score total	:	1.7
Score for star read(s)	:	-1.3
Score for read counts	:	0
Score for mfe	:	1.4
Score for randfold	:	1.6
Score for cons. seed	:	
Total read count	:	70
Mature read count	:	70
Loop read count	:	0
Star read count	:	0

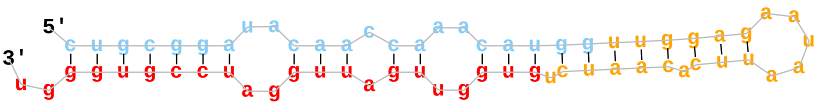

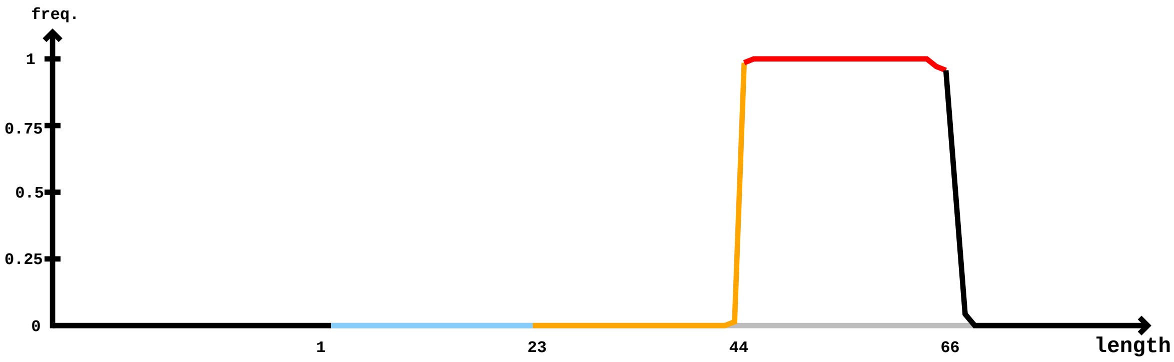

Star Mature

```
5'- agcccuccauaucugugguuucugcaccugcggauacaaccaaacaugguuggagaauaauucacaaucugugguugauuggauccguggguauagaaccugcaaauacaua   -3'    exp
    .........(((.(((((.(((((.(((((((((..(((.((..(((((((((((.....))).))))).)))..)).)))..))))))))).))))).))))).)))....     reads     mm     sample
    ....................................................................ugugguugauuggauccgugg.......................     1         0      seq
    .....................................................................gugguugauuggauccgugg.......................     1         0      seq
    .....................................................................gugguugauuggauccguggg......................     1         0      seq
    .....................................................................gugguugauuggauccgugggu.....................     56        0      seq
    .....................................................................gugguugauuggauccguggguа....................     8         0      seq
    .....................................................................gugguugauuggauccguggguau...................     2         0      seq
    ......................................................................ugguugauuggauccguggguau...................     1         0      seq
```
